# Prevalence of Myopia and Axial Length Distribution in China: The Wuhu Children and Adolescents Eye Study

**DOI:** 10.1167/iovs.66.6.33

**Published:** 2025-06-10

**Authors:** Li Dong, Wen-Da Zhou, Ya-Bin Hu, Li Wei, Wei Cao, Ning-Hong Chen, Di Liu, Cheng Zhen, Yu-Chun Zheng, Jost B. Jonas, Wen-Bin Wei

**Affiliations:** 1 Beijing Tongren Eye Center, Beijing Key Laboratory of Intraocular Tumor Diagnosis and Treatment, Beijing Ophthalmology and Visual Sciences Key Lab, Medical Artificial Intelligence Research and Verification Key Laboratory of the Ministry of Industry and Information Technology, Beijing Key Laboratory of Intelligent Diagnosis, Treatment and Prevention of Blinding Eye Diseases, Beijing Tongren Hospital, Capital Medical University, Beijing, China; 2 Mingsii Co., Ltd, Beijing, China; 3 Wuhu Center for Disease Control and Prevention, Wuhu, Anhui Province, China; 4 Department of Ophthalmology and Optometry, Ophthalmic Hospital of Wuhu, Wuhu, Anhui Province, China; 5 National Digital Health Center of China Top Think Tanks, Beijing Normal University, Beijing, China; 6 AIFuture Lab, Beijing, China; 7 Rothschild Foundation Hospital, Institut Français de Myopie, Paris, France; 8 Singapore Eye Research Institute, Singapore National Eye Center, Singapore; 9 Privatpraxis Prof. Jonas und Dr. Panda-Jonas, Heidelberg, Germany; 10 Beijing Visual Science and Translational Eye Research Institute (BERI), Beijing Tsinghua Changgung Hospital, Tsinghua Medicine, Tsinghua University, Beijing, China; 11 LV Prasad Eye Institute, Hyderabad, Telangana, India

**Keywords:** myopia, axial length, fundus tessellation, population-based study, childhood myopia

## Abstract

**Purpose:**

To re-assess myopia prevalence, update the database of ocular biometric parameters, and assess the prevalence of fundus abnormalities in schoolchildren and adolescents in Wuhu, Anhui Province, China.

**Methods:**

The Wuhu Children and Adolescents Eye Study (WCAES) is a longitudinal population-based cohort study, and the cross-sectional analysis results of the baseline assessment are reported here. It included 315,569 out of 330,173 eligible children and adolescents (95.6%), ages 2 to 19 years, from 513 schools with measurements of presenting distance visual acuity (PDVA) and uncorrected distance visual acuity (UDVA), non-cycloplegic autorefractometry, ocular biometry, and color fundus photography. Fundus abnormalities were assessed by an artificial intelligence–based system.

**Results:**

Prevalences of likely myopia (−1.00 D < refractive error ≤ −0.50 D), myopia (refractive error ≤−1.00 D), low myopia (−3.00 D < refractive error ≤ −1.00 D), moderate myopia (−6.00 D < refractive error ≤ −3.00 D), and high myopia (refractive error ≤ −6.00 D) were 13.58% (95% confidence interval [CI], 13.46–13.70), 56.92% (95% CI, 56.74–57.10), 32.87% (95% CI, 32.70–33.04), 19.74% (95% CI, 19.60–19.88), and 4.31% (95% CI, 4.24–4.38), respectively. Among high-school students, myopia prevalence was 92.18% (95% CI, 91.68–92.68). High myopia prevalence rates in kindergarten, elementary, middle, and high schools were 0.30% (95% CI, 0.18–0.43), 1.53% (95% CI, 1.48–1.59), 9.05% (95% CI, 8.86–9.24), and 18.57% (95% CI, 17.84–19.29), respectively. Higher prevalence of overall myopia and high myopia was associated (all *P* < 0.001) with female sex (odds ratio [OR] = 0.81 and OR = 0.91, respectively), older age (OR = 1.45 and OR = 1.48, respectively), longer axial length (OR = 3.71 and OR = 5.89, respectively), and higher prevalence of fundus tessellation (OR = 1.10 and OR = 1.21, respectively.). The percentage of myopic individuals using vision correction and the prevalence of an age-dependent defined suboptimal PDVA in the myopic population were 48.05% (95% CI, 47.81–48.28) and 65.84% (95% CI, 65.51–66.16), respectively.

**Conclusions:**

Compared with previous studies, the present investigation suggests a further rise in the prevalence of myopia, particularly high myopia, in the younger generations in China.

Refractive errors such as myopia, hyperopia, and astigmatism are the most common ocular disorders worldwide.^1^^,^^2^ It has been estimated that, by 2050, about half of the global population may have myopia, with approximately 10% of the global population being highly myopic.^2^^,^^3^ In China, myopia has emerged as a significant public health issue among the younger generations and, in future, with today's children and adolescents growing older, for the total population.^4^^,^^5^ In the last 20 years, the prevalence of myopia in children and adolescents in China has continuously increased, particularly after the COVID-19 pandemic, with the prevalence of myopia in children increasing by 9.5% to 15.8%.^6^ A meta-analysis estimated that the myopia prevalence in 2050 among children and adolescents ages 3 to 19 years might reach 84%,^7^ resulting in a marked increase in the incidence and prevalence of myopia-related diseases such as rhegmatogenous retinal detachment, open-angle glaucoma, myopic macular degeneration, and high myopia associated optic neuropathies.^8^^,^^9^ Developing nomograms for refractive error development and axial ocular growth from early childhood to early adulthood is clinically essential to estimate the further progression of myopia in the individual child, in particular to foresee at which value of myopic refractive error the progression may eventually end in adulthood.

Previous epidemiological studies have usually involved relatively small populations, ranging in size from several hundred to usually a few thousand participants.^10^^,^^11^ In some of these investigations, the study populations were not fully representative of the population they were aimed for, as the studies covered only a relatively small range of age of the study participants or the study regions were not fully representative for the intended geographical areas.^12^^,^^13^ To cite examples, the variation in myopia prevalence between urban regions and rural areas was not taken into account; also, in some studies axial length (AL) was not measured and refractometry was not performed under cycloplegic conditions.^14^^–^^16^ AL is an important parameter for evaluating the development of children's eyes.^17^ Because AL and corneal refractive power are the two most important biometric parameters for the refractive error of the eye, AL has been incorporated into the AL-to-corneal radius of curvature (AL/CRC) ratio. This ratio has been used as an alternative parameter for refractive error for diagnosing myopia and assessing its progression.^17^^,^^18^

In view of the limitations of some of the previous studies, and because it is important to reassess and to update the information of myopia prevalence, we initiated the longitudinal Wuhu Children and Adolescents Eye Study (WCAES), which included a large and representative sample of children and adolescents in Wuhu City, Anhui Province, China. The WCAES was designed as a longitudinal cohort study; baseline data collection was completed in May 2023, and the next follow-up examinations began in September 2023. The present study is based on the cross-sectional analysis results of the baseline assessment. The study goals were to reassess and update current information on the prevalence of myopia, particularly high myopia, in schoolchildren in China and to develop nomograms for predicting myopia progression in individual participants based on associated factors such as age, sex, region, and school grade. The study also evaluated the distribution of ocular biometric parameters, including AL, CRC, and the AL/CRC ratio and their associations. In addition, the investigation assessed prevalence and associated factors of fundus abnormalities in children and adolescents, with a particular focus on fundus tessellation.

## Methods

### Study Area and Population

The WCAES is a population-based study including children and adolescents in Wuhu City, Anhui Province, China. The ethics committee of the Beijing Tongren Hospital, Capital Medical University, Beijing, approved the study design, which adhered to the tenets of the Declaration of Helsinki. The project began with the Wuhu Education Bureau notifying the schools about the study, followed by the distribution of a promotional letter to parents by the Wuhu Education Bureau and the schools. The letter emphasized the importance of vision and eye disease screening and detailed the benefits and risks of this study.

The study was carried out in the city of Wuhu, a prefecture-level city located in southeastern Anhui Province of China on the Yangtze River. The total city area covers 6026 km^2^ with a permanent population of around 3.4 million residents (see Supplementary Table S1). Administratively, Wuhu consists of five districts (Jinghu, Jiujiang, Yijiang, Wanzhi, and Fanchang), one county-level city (Wuwei), and one county (Nanling). Wuhu is a significant industrial hub, with a focus on manufacturing, electronics, and the automotive industry. In 2023, its gross domestic product reached 381.5 billion RMB (or US$54.9 billion), making it the second-largest economy in Anhui Province, ranking 61st in China (see Supplementary Table S1). Led by the Wuhu Municipal Government, the Wuhu Municipal Health Commission, and the Wuhu Education Bureau, a study committee was established to oversee the organization, coordination, and supervision of the study. The committee's office was based at the Biomedical Big Data Center of the Beijing Tongren Hospital, Capital Medical University, Beijing.

### Study Design and Sampling Process

**Figure 1.**
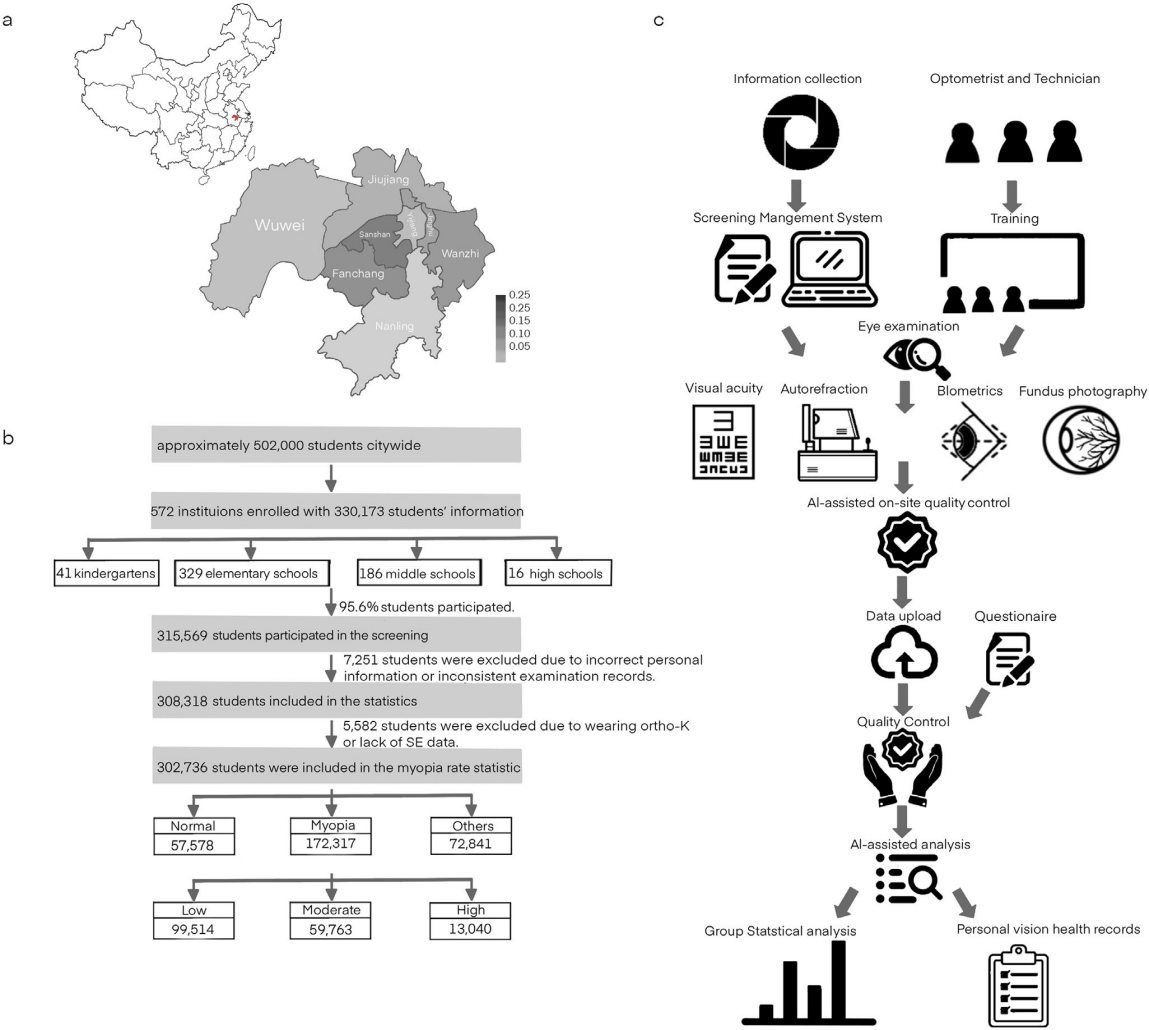
Flowchart of the WCAES. (**a**) Location of the WCAES and regional population percentage of Wuhu. (**b**) Framework of the WCAES. (**c**) Quality control, showing the participants available for analysis in the baseline.

The WCAES was designed as a school-based, longitudinal survey, and its baseline examination was conducted between February and May 2023. The study aimed to include all school-aged children and adolescents from elementary and middle schools throughout Wuhu, as well as kindergarten children and students at high schools in the two districts of Jinghu and Jiujiang of Wuhu. The study pursued four objectives: (1) to assess the prevalence of myopia and other refractive errors among children and adolescents in Wuhu to provide an update of myopia prevalence; (2) to determine ocular biometric parameters, including AL, CRC, and the AL/CRC ratio to identify potential biomarkers for myopia development; (3) to investigate the prevalence of fundus abnormalities in myopic children through color fundus photography and to explore their association with myopia; (4) to examine longitudinally the progression of refractive error, in particular myopia and high myopia, by conducting follow-up examinations; and (5) to examine risk factors influencing the onset and progression of myopia in children (Fig. 1).

The examinations were performed by 13 teams each consisting of 10 to 12 individuals, including optometrists, technicians, an on-site supervisor, and a school coordinator. Optometrists and technicians conducted the ophthalmic examinations. The optometrists were recruited from medical institutions and optometry clinics in Wuhu and possessed appropriate professional qualifications. Before the start of the study, all examiners underwent 2 days of operational standard training and hands-on equipment training, and they had to pass a final test.

Vocational schools and technical schools were not included in this study. The study population of the WCAES consisted of 330,173 individuals out of approximately 467,000 students in Wuhu city. The 330,173 individuals were from 572 institutions: 41 kindergartens (*n* = 9146, 2.8%), 329 primary schools (*n* = 211,397, 64.0%), 186 junior high schools (*n* = 94,068, 28.5%), and 16 senior high schools (*n* = 15,562, 4.7%) (see Supplementary Fig. S1). Based on the statistical results of education in Wuhu City for 2023 determined by the Wuhu Education Bureau, the students from vocational and technical schools accounted for about 7.4% (36,962 out of 502,000) of the total children and adolescent students in Wuhu city.^19^

A structured questionnaire was designed to collect information on potential risk factors for myopia. The questionnaire covered various domains, including demographic characteristics (age, sex, and place of residence), lifestyle and visual habits (daily routines, near-work activities, and screen-time exposure), and genetic predisposition (parental myopia history). The questionnaire was administered through parental proxy reporting using a web-based platform.

### Ophthalmic Examination

Presenting distant visual acuity (PDVA) and uncorrected distant visual acuity (UDVA) were measured for all participants using a Chinese standard visual acuity E chart (GB 11533-2011; Guangxi Anxin Tong Medical Equipment, Nanning, China). PDVA was assessed while the individual wore their current vision correction, if applicable. Individuals using orthokeratology lenses were excluded from the measurement of PDVA due to the potential influence of corneal reshaping on refractive measurements. The number and demographic characteristics of the excluded participants are presented in Supplementary Table S7. The study participants underwent assessment of non‑cycloplegic autorefractometry (FR-710 Autorefractometer; Ningbo FLO Optical Technology Development, Ningbo, China), with each eye being measured three times and the average value taken for further analysis. Children 6+ years old underwent ocular biometry with a SW-9000 optical biometer (Tianjin Suowei Electronic Technology, Tianjin, China) or FAL-2000A non-contact biometer (Hunan Huoyan Medical Technology, Xiangtan, China), with each measurement being repeated five times per eye and the average value calculated. Also, children 6+ years old underwent 45° color fundus photography, centered on the fovea (Kestrel 3100m non-mydriatic fundus camera; Chongqing Bio New Vision Medical Equipment, Chongqing, China). Each eye was imaged twice, and the photographic quality was assessed immediately after the images were taken. All measurements were reviewed by a senior examiner, and when there were doubts about the results, the measurements were repeated.

### Definitions

Visual acuity was assessed monocularly. Suboptimal visual acuity was defined depending on age according to the criteria of the National Health Commission of the People's Republic of China.^20^ UDVA or PDVA was suboptimal if the visual acuity was >0.3 logarithm of the minimal angle of resolution (logMAR) for children under 4 years old, if visual acuity was >0.22 logMAR for children 4 years old, or if the visual acuity was >0.00 logMAR for children 5+ years old.

Because refractometry was performed under non-cycloplegic conditions, we defined likely myopia as a refractive error range of >−1.00 diopter (D) and ≤−0.50 D in at least one eye, and myopia by a refractive error of ≤−1.00 D in at least one eye.^21^ Myopia was further differentiated into low myopia (−3.00 D < refractive error ≤ −1.00 D), moderate myopia (−6.00 D < refractive error ≤ −3.00 D), and high myopia (refractive error ≤ −6.00 D). Hyperopia was defined as a refractive error of ≥+2.00 D in any eye, astigmatism as a cylindric refractive error of ≤−0.75D in any eye, and anisometropia as an inter-eye difference in refractive error (spherical equivalent) of ≥1.50 D.

Using the biometric AL measurements, we defined high axial myopia as an AL of ≥26 mm. The AL/CRC ratio was defined as AL divided by the mean CRC. The mean corneal refractive (CR) power was the mean of the CR values measured in the two perpendicular meridians of the corneal astigmatism. The CRC was deduced from the mean CR power using the formula CRC = 0.3375/mean CR power (D) × 1000. The mean AL and mean AL/CRC were calculated based on the data of the eye with longer AL or larger AL/CRC. The mean CRC calculation was based on the corneal measurements obtained from the eye demonstrating the larger AL/CRC ratio.

### Artificial Intelligence System

We applied a retinal artificial intelligence (AI)-based system for the detection of fundus abnormalities on the fundus photographs.^22^^,^^23^ The retinal AI system consisted of a first component utilizing a detection model to identify the macula and optic nerve head and a second component featuring a multitask convolutional neural network with three subtasks to identify fundus abnormalities. The subtasks processed inputs from the whole fundus image, the macular region, and the optic nerve head region, respectively. The system was capable of detecting 11 fundus abnormalities, including fundus tessellation, an abnormally high cup-to-disc diameter ratio (i.e., a ratio of >0.6 as a surrogate of a large optic disc), epiretinal membranes, retinal detachment, macular hole, pathological myopia (defined as diffuse chorioretinal atrophy or greater degree of atrophy), retinal vein occlusions, diabetic retinopathy, optic disc edema, optic nerve atrophy, and retinal arteriosclerosis. Representative examples of fundus abnormalities are shown in Supplementary Figure S3. In our internal validation using a multicenter dataset of 8758 fundus images, the model showed a high diagnostic performance across all detected retinal diseases, with an accuracy exceeding 95% for each condition (own unpublished data). The area under the receiver operating characteristic curve (AUC) ranged from 0.93 to 0.99, indicating excellent discriminatory ability. For quality assurance, all fundus photographs diagnosed as positive by the AI system underwent a secondary review by two experienced retinal specialists. In cases of disagreement, the images were further assessed by a senior retinal specialist for a final consensus.

For data quality control, we used an AI-assisted data quality control system. It was developed based on the examination reports from more than 20,000 individuals whose data were manually assessed and controlled. Its accuracy was continuously improved by incorporating new data as the study progressed.^23^ In a first step, the AI data quality control system eliminated null values and data that exceeded the detection range of the equipment or theoretical limits. Subsequently, the system comprehensively analyzed various examination results to detect conflicts or inconsistencies between different results, such as a refractive error of <−3.0 D but an uncorrected visual acuity of >0.0 logMAR. In the third step, the system automatically identified anomalies in the data based on historical data and manual quality control results. It then induced either an elimination of the data or a review, depending on the type of anomaly. For data the system deemed questionable, optometrists conducted a comprehensive assessment of all examination data of the study participants and decided about inclusion of the data into the further analysis. In addition, all images for which the retinal AI system detected a fundus abnormality were reviewed by ophthalmologists who were specialized in retinal diseases.

### Statistical Analysis

Statistical analyses were performed using SPSS Statistics 27.0 (IBM, Chicago, IL, USA). The prevalence of myopia was calculated based on data from the eye with the more myopic refractive error or the longer AL, with the total number of individuals as the denominator. For fundus abnormalities, an individual was classified as having fundus abnormalities if at least one eye exhibited any abnormality. The prevalence of fundus abnormalities was analyzed using the total number of individuals as the denominator. All parameters were tested for normal distribution using the Shapiro–Wilk method. Data that met the criteria for normal distribution were expressed as mean ± standard deviation (SD), and overall differences between groups were compared using Student's *t*-test for unpaired samples and by a one-way analysis of variance (ANOVA). Data with a skewed distribution were expressed as the median (quartile 1 [Q1], quartile 3 [Q3]), and intergroup comparisons were performed using the Mann–Whitney test. Intergroup differences in categorical data were examined applying the χ^2^ test. Univariable and multivariable logistic regression analyses were conducted to further explore the relationships between potential risk factors and prevalence. Variables that demonstrated a statistically significant association in univariable analyses were subsequently included in the multivariable regression model. All confidence intervals (CIs) were given as 95% intervals. A two-tailed test was used, and *P* < 0.05 was considered statistically significant.

## Results

### Refractive Error

Out of 330,173 eligible individuals, 315,569 of the children and adolescents (95.6%) participated in the study. After data quality screening, the examination results obtained from 302,736 students (91.7% of the eligible population) were eventually included in the statistical analysis. The mean age of the 162,366 boys (53.63%) and 140,370 girls (46.37%) was 10.6 ± 2.9 years (range, 2–19). The exclusion was mainly due to incomplete or incorrect personal information and inconsistencies in examination records caused by systematic errors during initial data collection. Additionally, participants were excluded if their myopia status could not be determined due to the use of orthokeratology lenses or the absence of spherical equivalent data. A comparison between excluded and included participants is provided in Supplementary Table S7.

**Figure 2.**
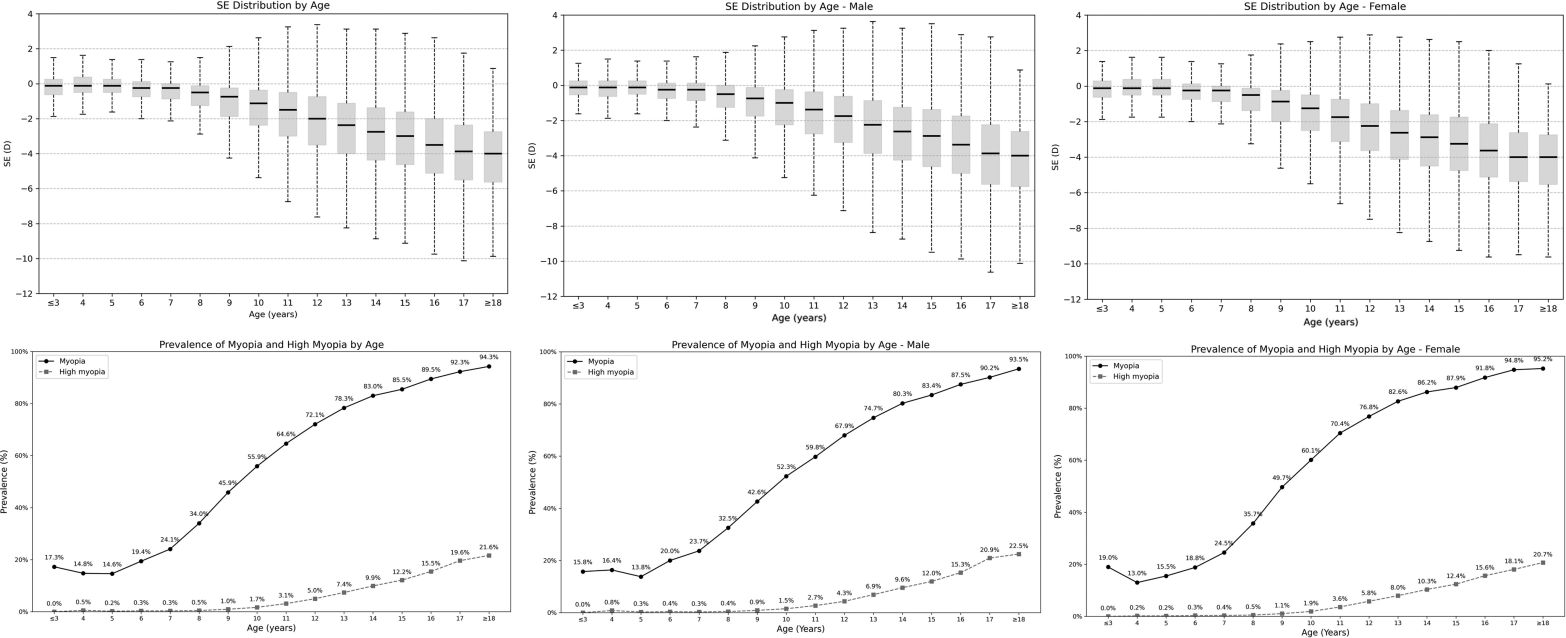
Distribution of refractive error and prevalence of myopia (defined as a refractive error ≤ −1.00 D) for children and adolescents in the WCAES, stratified by age and sex.

The overall prevalence of likely myopia, myopia, low myopia, moderate myopia, and high myopia was 13.58% (95% CI, 13.46–13.70), 56.92% (95% CI, 56.74–57.10), 32.87% (95% CI, 32.70–33.04), 19.74% (95% CI, 19.60–19.88), and 4.31% (95% CI, 4.24–4.38), respectively (Supplementary Table S2, Fig. 2^3^). The prevalence of myopia was 14.14% (95% CI, 13.31–14.93) in kindergarten, 45.73% (95% CI, 45.51–45.95) in elementary schools, 81.18% (95% CI, 80.92–81.45) in middle schools, and 92.18% (95% CI, 91.68–92.68) in high schools. High myopia prevalence in kindergarten, elementary, middle, and high schools was 0.30% (95% CI, 0.18–0.43), 1.53% (95% CI, 1.48–1.59), 9.05% (95% CI, 8.86–9.24), and 18.57% (95% CI, 17.84–19.29), respectively. In univariable analysis, a higher prevalence of myopia and high myopia and a higher degree of myopia were significantly (*P* < 0.001) associated with female sex, older age, higher school grade, longer AL, and higher prevalence of fundus tessellation (Supplementary Table S8).

**Figure 3.**
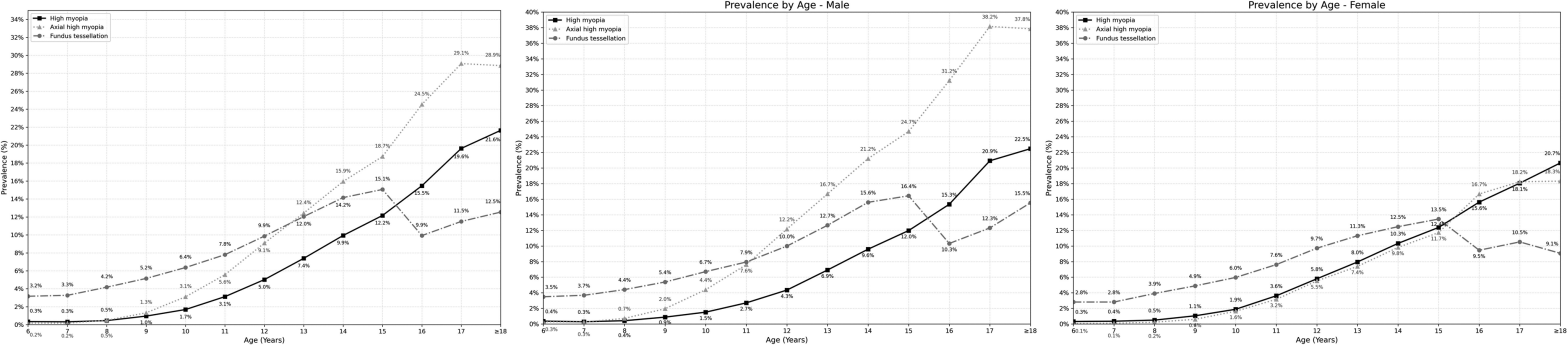
Prevalence of high myopia (defined as a refractive error ≤ −6.00 D), high axial myopia (defined by an AL of ≥26.0 mm), and fundus abnormalities in the WCAES stratified by age and sex.

In multivariable analysis, higher prevalence of overall myopia was associated with female sex (OR = 0.37; 95% CI, 0.37–0.38; *P* < 0.001), older age (OR = 1.20; 95% CI, 1.20–1.21; *P* < 0.001), longer AL (OR = 3.71; 95% CI, 3.66–3.75; *P* < 0.001), urban versus rural region of habitation (OR = 1.06; 95% CI, 1.02–1.10; *P* < 0.001), and higher prevalence of fundus tessellation (OR = 1.10; 95% CI, 1.05–1.15; *P* < 0.001). Higher prevalence of likely myopia was associated with younger age (OR = 0.96; 95% CI, 0.96–0.97; *P* < 0.001), male sex (OR = 1.38; 95% CI, 1.35–1.41; *P* < 0.001), rural versus urban region of habitation (OR = 0.94; 95% CI, 0.89–0.98; *P* < 0.001), longer AL (OR = 0.65; 95% CI, 0.64–0.65; *P* < 0.001), and higher prevalence of fundus tessellation (OR = 0.85; 95% CI, 0.81–0.89; *P* < 0.001). Higher prevalence of high myopia was associated with female sex (OR = 0.32; 95% CI, 0.31–0.34; *P* < 0.001), older age (OR = 1.12; 95% CI, 1.10–1.13; *P* < 0.001), longer AL (OR = 5.89; 95% CI, 5.73–6.05; *P* < 0.001), and higher prevalence of fundus tessellation (OR = 1.21; 95% CI, 1.14–1.28; *P* < 0.001) (Supplementary Table S8).

The mean UDVA and mean PDVA were 0.2 logMAR (Q1 0.00, Q3 0.60) and 0.10 logMAR (Q1 0.00, Q3 0.20), respectively (Table 1). The prevalence of suboptimal UDVA was 40.11% (95% CI, 38.96–41.26) in kindergartens, 55.89% (95% CI, 55.67–56.11) in elementary schools, 83.36% (95% CI, 83.11–83.61) in middle schools, and 93.58% (95% CI, 93.11–94.03) in high schools, respectively (Supplementary Table S3). In univariable analysis, a higher percentage of suboptimal UDVA was significantly (*P* < 0.001) associated with female sex, older age, higher school grade, longer AL, larger AL/CRC ratio, urban versus rural region of habitation, and higher degree of fundus tessellation. In multivariable analysis, a higher prevalence of suboptimal UDVA was associated with female sex (OR = 0.61; 95% CI, 0.60–0.62; *P* < 0.001), older age (OR = 1.09; 95% CI, 1.08–1.09; *P* < 0.001), longer AL (OR = 1.67; 95% CI, 1.65–1.69; *P* < 0.001), urban versus rural region of habitation (OR = 1.14; 95% CI, 1.10–1.19; *P* < 0.001), higher prevalence of fundus tessellation (OR = 1.20; 95% CI, 1.15–1.25; *P* < 0.001), and higher prevalence of myopia (OR = 6.07; 95% CI, 5.94–6.20; *P* < 0.001)

**Table 1.** Mean AL, Prevalence of High Axial Myopia, Mean Presenting Distant Visual Acuity, and Mean Uncorrected Distant Visual Acuity in the WCAES, Stratified by Sex, Region, and Age

	*N*	AL (mm), Mean ± SD	Prevalence of High Axial Myopia (AL > 26 mm), % (95% CI)	Presenting Distant Visual Acuity (logMAR), Median (Q1, Q3)	Uncorrected Distant Visual Acuity (logMAR), Median (Q1, Q3)
Sex					
Male	154,546	24.28 ± 1.24	9.59 (9.44–9.73)	0.10 (0.00, 0.20)	0.20 (0.00, 0.60)
Female	134,184	23.79 ± 1.22	4.26 (4.16–4.37)	0.10 (0.00, 0.20)	0.20 (0.00, 0.70)
Region					
Urban	271,454	24.06 ± 1.26	7.18 (7.09–7.28)	0.10 (0.00, 0.20)	0.20 (0.00, 0.60)
Rural	17,276	23.98 ± 1.19	6.04 (5.70–6.40)	0.10 (0.00, 0.30)	0.20 (0.00, 0.60)
Age (y)					
6	13,504	22.87 ± 0.84	0.19 (0.12–0.26)	0.00 (0.00, 0.10)	0.00 (0.00, 0.10)
7	30,133	23.03 ± 0.87	0.18 (0.13–0.23)	0.00 (0.00, 0.10)	0.00 (0.00, 0.10)
8	33,355	23.37 ± 0.92	0.49 (0.42–0.57)	0.00 (0.00, 0.10)	0.00 (0.00, 0.20)
9	31,739	23.70 ± 0.97	1.33 (1.21–1.45)	0.00 (0.00, 0.20)	0.10 (0.00, 0.30)
10	33,650	23.98 ± 1.04	3.11 (2.93–3.29)	0.10 (0.00, 0.20)	0.20 (0.00, 0.50)
11	31,356	24.23 ± 1.09	5.57 (5.32–5.83)	0.10 (0.00, 0.30)	0.30 (0.00, 0.60)
12	28,697	24.45 ± 1.13	9.08 (8.75–9.42)	0.10 (0.00, 0.30)	0.40 (0.10, 0.80)
13	28,643	24.64 ± 1.17	12.43 (12.04–12.82)	0.10 (0.00, 0.30)	0.50 (0.10, 0.90)
14	27,891	24.80 ± 1.21	15.95 (15.53–16.38)	0.10 (0.00, 0.30)	0.60 (0.20, 1.00)
15	18,500	24.90 ± 1.24	18.74 (18.18–19.30)	0.10 (0.00, 0.30)	0.70 (0.30, 1.00)
16	5,822	25.10 ± 1.31	24.53 (23.45–25.61)	0.10 (0.00, 0.20)	0.80 (0.40, 1.00)
17	3,838	25.28 ± 1.28	29.08 (27.64–30.48)	0.10 (0.00, 0.20)	0.80 (0.50, 1.00)
≥18	1,601	25.33 ± 1.30	28.86 (26.67–31.04)	0.10 (0.10, 0.30)	0.90 (0.60, 1.00)

The percentage of myopic individuals using vision correction and the prevalence of suboptimal PDVA in the myopic population were 48.05% (95% CI, 47.81–48.28) and 65.84% (95% CI, 65.51–66.16), respectively. The percentage of myopic individuals using vision correction in urban areas was significantly higher than that in rural areas (*P <* 0.001), whereas the percentage of suboptimal PDVA in the myopic population was significantly lower in urban areas than in rural areas (*P* < 0.001) (Supplementary Table S3). In univariable analysis, a higher prevalence of suboptimal PDVA in the myopic population was significantly (*P* < 0.001) associated with female sex, older age, higher school grade, longer AL, larger AL/CRC ratio, and higher degree of fundus tessellation. In multivariable analysis, a higher percentage of suboptimal PDVA in the myopic population was associated with female sex (OR = 0.49; 95% CI, 0.48–0.51; *P* < 0.001), older age (OR = 1.12; 95% CI, 1.11–1.12; *P* < 0.001), longer AL (OR = 2.22; 95% CI, 2.19–2.25; *P* < 0.001), urban versus rural region of habitation (OR = 1.31; 95% CI, 1.25–1.38; *P* < 0.001), and higher prevalence of fundus tessellation (OR = 1.07; 95% CI, 1.03–1.11; *P* < 0.001).

### Axial Length

After data quality screening, valid AL data were obtained for 288,730 individuals (87.4% of the eligible population; 154,546 [53.53%] boys; 577,254 eyes) with a mean age of 10.73 ± 2.83 years (range, 6–19) and a mean AL of 24.05 ± 1.26 mm. Table 1 shows the mean AL and prevalence of high axial myopia stratified by sex, region, and age.

The prevalence of high axial myopia was higher in boys than girls (9.59% vs. 4.26%; *P* < 0.001) and was more common in older age groups up to 17 years of age, in individuals with a higher school grade (*P* < 0.001), and in urban compared to rural regions (*P* < 0.001) (Fig. 3). In multivariable analysis, a higher prevalence of high axial myopia was significantly associated with older age (OR = 1.19; 95% CI, 1.18–1.20; *P* < 0.001), male sex (OR = 2.97; 95% CI, 2.85–3.09; *P* < 0.001), urban versus rural region of habitation (OR = 1.12; 95% CI, 1.03–1.21; *P* < 0.001), and higher prevalence of fundus tessellation (OR = 2.16; 95% CI, 2.06–2.26; *P* < 0.001) (see Supplementary Table S8).

**Figure 4.**
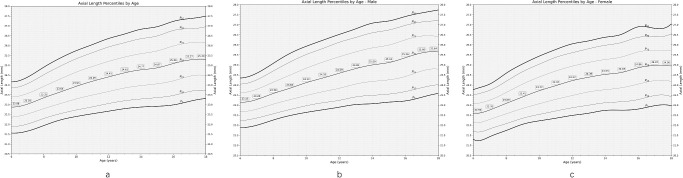
AL percentiles in the WCAES stratified by age and sex.

Supplementary Table S4 shows the percentiles of AL stratified by age, region, and sex. The AL measurements expressed in percentiles were greater in the older age group. The amount of enlargement in the AL measurements gradually slowed down beyond the age of 16 years (Fig. 4). The range of the percentile curves grew broader with older age.

### CRC and AL/CRC Ratio

After data quality screening, AL/CRC ratio data were obtained from 287,905 individuals (87.2% of the eligible population; 154,096 [53.52%] boys; 571,430 eyes). The mean age was 10.73 ± 2.83 years (range, 6–19). The mean CRC value and mean AL/CRC ratio were 7.83 ± 0.27 and 3.07 ± 0.15, respectively. Table 2 shows the mean CRC and mean AL/CRC ratio stratified by sex, region, and age. The AL/CRC ratio was higher in boys (3.07 ± 0.15 for boys vs. 3.06 ± 0.15 for girls; *P* < 0.001) and higher in the urban region of habitation (3.07 ± 0.15 for urban vs. 3.06 ± 0.14 for rural; *P* < 0.001). The CRC was larger in boys (7.89 ± 0.27 for boys vs. 7.77 ± 0.26 for girls; *P* < 0.001) and larger in the urban region of habitation (7.83 ± 0.27 for urban vs. 7.82 ± 0.27 for rural; *P* < 0.001).

**Table 2.** CRC Values and AL/CRC Ratios in the WCAES, Stratified by Sex, Region, and Age

	*N*	CRC Value (mm), Mean ± SD	AL/CRC Ratio, Mean ± SD
Sex			
Male	154,096	7.891 ± 0.266	3.07 ± 0.15
Female	133,809	7.766 ± 0.259	3.06 ± 0.15
Region			
Urban	270,640	7.834 ± 0.270	3.067 ± 0.151
Rural	17,263	7.823 ± 0.269	3.062 ± 0.142
Age (y)			
6	13,467	7.802 ± 0.267	2.93 ± 0.10
7	30,013	7.803 ± 0.267	2.95 ± 0.10
8	33,212	7.822 ± 0.270	2.98 ± 0.11
9	31,686	7.832 ± 0.269	3.02 ± 0.11
10	33,625	7.841 ± 0.270	3.05 ± 0.12
11	31,321	7.840 ± 0.273	3.09 ± 0.13
12	28,620	7.843 ± 0.271	3.11 ± 0.14
13	28,497	7.850 ± 0.270	3.14 ± 0.14
14	27,788	7.844 ± 0.267	3.16 ± 0.15
15	18,454	7.841 ± 0.267	3.17 ± 0.15
16	5,801	7.839 ± 0.275	3.20 ± 0.16
17	3,827	7.832 ± 0.277	3.22 ± 0.16
≥18	1,593	7.821 ± 0.278	3.23 ± 0.16

### Fundus Abnormalities

After data quality screening, images were obtained from 286,858 individuals (86.9% of the eligible population; 153,590 [53.54%] boys; 573,714 eyes) with a mean age of 10.74 ± 2.83 years (range, 6–19). All photographs were examined by the AI system for fundus abnormalities and subsequently reviewed by a clinical expert. Table 3 shows the prevalence of fundus abnormalities stratified by age, region, and sex. The most common fundus abnormalities detected were fundus tessellation and an increased cup-to-disc ratio of the optic nerve head (or large optic disc). Other fundus abnormalities included macular epiretinal membrane (*n* = 19 eyes), retinal arteriosclerosis (*n* = 19 eyes), retinal detachment (*n* = 5 eyes), pathological myopia (*n* = 4 eyes), optic nerve atrophy (*n* = 3 eyes), retinal vein occlusion (*n* = 3 eyes), macular hole (*n* = 3 eyes), optic disc edema (*n* = 1 eye), and diabetic retinopathy (*n* = 1 eye) (Supplementary Table S6). The prevalence of fundus tessellation was 8.07% (95% CI, 7.97–8.17) and was higher in boys than in girls (8.60% vs. 7.45%; *P* < 0.001) (Fig. 3). In univariable analyses, a higher prevalence of fundus tessellation was associated with older age, (OR = 1.19; 95% CI, 1.19–1.20; *P* < 0.001), male sex (OR = 1.17; 95% CI, 1.14–1.20; *P* < 0.001), higher school grade (OR = 1.21; 95% CI, 1.20–1.21; *P* < 0.001), urban versus rural habitation (OR = 1.89; 95% CI, 1.76–2.03; *P* < 0.001), longer AL (OR = 1.82; 95% CI, 1.80–1.84; *P* < 0.001), larger AL/CRC ratio (OR = 48.1; 95% CI, 44.1–52.4; *P* < 0.001), and higher prevalence of myopia (OR = 2.78; 95% CI, 2.69–2.87; *P* < 0.001) (Supplementary Fig. S2). In multivariable analysis, a higher prevalence of fundus tessellation was significantly associated with male sex (OR = 1.22; 95% CI, 1.19–1.25; *P* < 0.001), older age (OR = 1.14; 95% CI, 1.14–1.15; *P* < 0.001), urban versus rural region of habitation (OR = 1.98; 95% CI, 1.84–2.13; *P* < 0.001), and higher prevalence of myopia (OR = 2.02; 95% CI, 1.95–2.09; *P* < 0.001) (Supplementary Table S8). The overall prevalence of fundus tessellation was 6.16% in eyes, ranging from 2.99% in eyes without myopia, to 3.98% in eyes with likely myopia, 6.01% in eyes with low myopia, 11.85% in eyes with moderate myopia, and to 21.18% in highly myopic eyes.

**Table 3.** Prevalence of Fundus Abnormalities in the WCAES, Stratified by Sex, Region, and Age

	*N*	Fundus Tessellation, % (95% CI)	Increased Cup-to-Disc Ratio, % (95% CI)	Other, % (95% CI)
Sex				
Male	153,590	8.60 (8.46–8.74)	2.09 (2.02–2.17)	0.0156 (0.0098–0.0221)
Female	133,270	7.45 (7.31–7.60)	1.31 (1.25–1.37)	0.0090 (0.0045–0.0143)
Region				
Urban	269,218	8.30 (8.19–8.40)	1.72 (1.67–1.77)	0.0123 (0.0082–0.0163)
Rural	17,642	4.56 (4.27–4.86)	1.87 (1.68–2.07)	0.0170 (0.0000–0.0397)
Age (y)				
6	13,350	3.17 (2.88–3.47)	1.92 (1.70–2.15)	0.0000 (0.0000–0.0000)
7	29,886	3.27 (3.07–3.48)	2.13 (1.98–2.30)	0.0067 (0.0000–0.0167)
8	33,019	4.18 (3.97–4.39)	2.10 (1.95–2.26)	0.0182 (0.0061–0.0363)
9	31,431	5.15 (4.92–5.40)	1.74 (1.59–1.89)	0.0064 (0.0000–0.0159)
10	33,294	6.37 (6.10–6.63)	1.75 (1.61–1.89)	0.0060 (0.0000–0.0150)
11	31,223	7.80 (7.50–8.09)	1.51 (1.38–1.65)	0.0064 (0.0000–0.0160)
12	28,644	9.86 (9.53–10.21)	1.53 (1.39–1.68)	0.0140 (0.0035–0.0279)
13	28,810	12.04 (11.66–12.41)	1.56 (1.41–1.70)	0.0069 (0.0000–0.0174)
14	27,826	14.16 (13.77–14.56)	1.54 (1.40–1.69)	0.0216 (0.0072–0.0395)
15	18,276	15.06 (14.55–15.59)	1.53 (1.35–1.71)	0.0219 (0.0055–0.0438)
16	5,723	9.92 (9.16–10.73)	1.73 (1.40–2.08)	0.0349 (0.0000–0.0874)
17	3,782	11.50 (10.50–12.53)	1.37 (1.00–1.75)	0.0793 (0.0000–0.1851)
≥18	1,595	12.54 (10.91–14.17)	1.38 (0.82–2.01)	0.0627 (0.0000–0.1881)

The prevalence of an abnormally high cup-to-disc diameter ratio was 1.73% (95% CI, 1.68–1.78). It was related with male sex (2.09% for boys vs. 1.31% for girls; *P* < 0.001), lower school grade (*P* < 0.001), younger age (*P* < 0.001), longer AL (*P* < 0.001), larger AL/CRC ratio (*P* < 0.001), higher prevalence of fundus tessellation (*P* < 0.001), and lower prevalence of myopia (*P* < 0.001). The prevalence of an abnormally high cup-to-disc diameter ratio in elementary schools, middle schools and high schools was 1.82% (95% CI, 1.76–1.87), 1.57% (95% CI, 1.49–1.66) and 1.42% (95% CI, 1.21–1.64), respectively (Supplementary Table S5). In multivariable analysis, a higher prevalence of a high cup-to-disc ratio was significantly associated with male sex (OR = 1.46; 95% CI, 1.38–1.56; *P* < 0.001), younger age (OR = 0.94; 95% CI, 0.93–0.95; *P* < 0.001), longer AL (OR = 1.15; 95% CI, 1.11–1.19; *P* < 0.001), higher prevalence of fundus tessellation (OR = 2.12; 95% CI, 1.95–2.31; *P* < 0.001), and lower prevalence of myopia (OR = 0.69; 95% CI, 0.64–0.74; *P* < 0.001) (Supplementary Table S8).

## Discussion

To the best of our knowledge, the WCAES features the largest population-based database of ocular biometric parameters in schoolchildren and adolescents, including the parameters of AL and AL/CRC ratio, among existing global epidemiological studies on myopia. In this large school-based study conducted in a medium-sized city in southeastern China, the prevalence of myopia (mean, 56.9%) was lowest in kindergarten children (14.1%) and highest among high-school students (92.2%). As a corollary, the prevalence of high myopia (overall mean, 4.3%) was lowest in kindergarten (0.3%) and highest in high school (18.6%). Both prevalence rates were associated with female sex, older age, and urban versus rural region of habitation. The overall prevalence of fundus tessellation was 8.07% per study participant and 6.16% per eye, and it was lowest in eyes without myopia (2.99%) and highest in highly myopic eyes (21.18%).

The results of the study fully support the findings obtained in previous investigations on the prevalence of myopia among the younger generations in China. In a recent meta-analysis covering the period from 1998 to 2016, the pooled prevalence rates of myopia and high myopia in individuals 3 to 19 years of age were 37.7% and 3.1%, respectively.^7^ The prevalence of myopia was strongly associated with older age, urban region of habitation, female sex, and higher study year. Before as compared to after 2008, myopia prevalence increased from 25.3% to 32.8% in 7- to 12-year-olds, and from 48.4% to 58.7% in 16- to 18-year-olds. Studies performed after 2013 showed prevalence rates of myopia and high myopia in those 16 to 18 years old of 84.8% (95% CI, 84.4–85.2) and 19.3% (95% CI, 18.6–20.2), respectively.^7^

Our study showed a continuing trend of further increasing prevalence rates of myopia and in particular of high myopia in the younger generations of China. Although the overall prevalence of myopia in the group of 18-year-olds cannot rise much more due the ceiling effect of a value of 100%, the prevalence of high myopia in the present study was approximately 19%, higher than in studies conducted 5 or 10 years ago.^7^ To cite examples, the population of the Gobi Children Eye Study, performed in 2013, had a prevalence of high myopia (defined by a refractive error of <−6 D) of 9.1% in those 17 to 18 years old.^24^ In the Shandong Children Eye Study, performed in 2012, the 17-year-olds had an overall prevalence of myopia of 84.6% ± 3.2% (95% CI, 78.0–91.0) and of high myopia of 13.9% ± 3.0% (95% CI, 7.8–19.9).^25^

Although moderate myopia confers certain benefits, including functional correction of presbyopia and a protective effect against age-related macular degeneration, diabetic retinopathy, and angle-closure glaucoma, it is high myopia that is the most important risk factor for myopic macular degeneration, and high myopia-related optic neuropathy has become one of the most common causes for irreversible vision impairment and blindness.^26^ The clinical importance of the findings obtained in our study is therefore based more on the increasing prevalence of high myopia than on the prevalence of moderate myopia. Although it has not been shown yet that the marked myopia among schoolchildren of today leads to pathologic myopia in adulthood, the risk of eventual pathologic changes in the retina and optic nerve of markedly myopic schoolchildren in their later life is definitely a given and increases with longer AL in previous investigations. Recent studies have reported that the risk of continuous axial elongation in myopic and highly myopic adults increased with longer AL at baseline.^8^^,^^27^ In the population-based Ural Very Old Study on individuals ages 85+ years, the prevalence of stage 3+ myopic macular degeneration was higher than 755 in eyes with AL of 26.5 mm or longer.^28^ If that study population is representative of other populations, its findings indicate that an individual with an ocular AL of more than 26.5 mm has a risk of 75% of developing a severe form of myopic macular degeneration beyond the age of 85 years. These observations suggest that measures should be taken to reduce myopia progression by procedures such as topical low-dose atropine eye-drop application or optical measures.^29^

The associations between a higher prevalence and amount of overall myopia and of high myopia with female sex, older age, and urban versus rural region of habitation as found in our study population agree with the observations made in previous investigations.^7^^,^^8^^,^^24^^–^^27^ Notably, our study found that the prevalence of myopia and high myopia was significantly higher in females than in males. However, when high myopia was defined based on AL (i.e., axial high myopia), the prevalence of axial high myopia was significantly higher in males than in females. Additionally, the AL/CRC ratio was significantly higher in males. This discrepancy could be primarily attributed to the longer AL in males, coupled with an overall weaker refractive power of their cornea and lens. Correspondingly, the CRC value was significantly higher in males than in females, indicating that males had a lower corneal refractive power in relationship to AL. In a parallel manner, previous studies reported that the refractive lens power was lower in males than in females.^30^ To arrive at the same refractive status, males as compared to females thus required a longer AL. Correspondingly, the prevalence of AL-dependent fundus tessellation was higher in males than in females.

Interestingly, the prevalence of fundus tessellation decreased after the age of 16 years, despite an ongoing axial elongation. The reason for the discrepancy may be that prevalence and degree of fundus tessellation depend on a multitude of parameters besides longer AL, such as older age, male sex, lower body mass index, worse best-corrected visual acuity, thinner subfoveal choroidal thickness, and larger parapapillary beta zone, among others, with subfoveal choroidal thickness being by far the most important parameter.^31^ In addition, post-pubertal age-related changes in the melanin content of the choroidal melanocytes and retinal pigment epithelium cells may also play a role.^32^ These factors may explain the decrease in the prevalence of fundus tessellation in individuals older than 16 years in our cohort, despite an ongoing axial elongation.

In addition to confirming trends in the prevalence of myopia as also reported in previous investigations, the novel results of the present study are based on several factors. The study area of Wuhu represents an economically developing region in China, with a development level lower than that of the southeastern coastal areas but higher than that of the less-developed northwestern regions. As an example of a rapidly industrializing region, the study may provide insights into the impact of rapid industrialization-related lifestyle changes on myopia prevalence, incidence, and overall visual health. Previous studies on the prevalence of childhood myopia were conducted mainly in metropolitan regions of the Pacific coast or in the less developed western areas of China. The present study is the most up-to-date assessment of schoolchildren myopia, interestingly revealing a further increase in myopia prevalence. The study includes the largest dataset collected so far on ocular biometric parameters including AL and corneal curvature in the Chinese school-aged population. It is also the largest population-based study on the prevalence and associations of fundus abnormalities in schoolchildren and describes their assessment by an AI-based method.

Limitations of our study should be discussed. First, despite its large sample size of more than 300,000 participants and despite its wide age range from 2 to 19 years, our study population was not representative of the entire young population of China. A strength of our study, however, is that it was not conducted in one of the very large and highly developed metropolitan regions of China at the Pacific coast but rather in the hinterland, thus representing a population between the highly developed China in the East and less developed regions in West China. Second, refractometry was not conducted in cycloplegia, so that accommodation during the measurement might have falsely increased the prevalence of myopia. Aware of this limitation, the examiners instructed the participants to relax their accommodation during the refractometry, which may have potentially reduced the effect of the potential bias and may hold true in particular for high myopia, in which the accommodative range is reduced. In addition, AL measurements are independent of the accommodative status, and the prevalence of axial myopia and high axial myopia was similar to the prevalence rates of myopia defined by the refractive error. Third, variations in the definition of myopia and high myopia between our study and previous investigations have to be taken into account when the results of different studies are compared with each other. The definition of high myopia with a myopic refractive error of more than −6 D in children and adolescents has, however, been applied in most previous studies, as well as in our investigation. Fourth, information about some factors reported to be associated with myopia, such as parental myopia, outdoor activity, time spent outdoors, and amount of near work, was not assessed in our study, so that the multivariable analyses conducted in our study were not complete with respect to the potentially full list of independent variables. Strengths of our study include its large sample size, the relative representativeness of the Wuhu region, the inclusion of urban and rural regions, and the timeliness of our study.

In conclusion, compared with the results of previously conducted investigations, the prevalence of myopia and high myopia has further increased in the young generation of China. In particular, the ongoing increase in the prevalence of high myopia as a major risk factor for pathologic myopia in adulthood may give rise to concern with respect to myopia-related vision impairment in the future in China, when the highly myopic schoolchildren of today have grown into adults in their mid-50s.

## Supplementary Material

Supplement 1
