# Supplementary material for: Prevalence of Myopia and Axial Length Distribution in China: The Wuhu Children and Adolescents Eye Study
Source: Invest Ophthalmol Vis Sci. 2025 Jun 10;66(6):33. doi: 10.1167/iovs.66.6.33 (PMC12165258; doi:10.1167/iovs.66.6.33)

**Table of Contents**

**Section S1: Supplementary eTable 1-8:**

**Supplementary eTable 1.** Gross Regional Product（GDP）and Resident Population of Wuhu City by District and County, 2023……………………………………………………………………….….…..2

**Supplementary eTable 2.** Prevalence of myopia and high myopia in the Wuhu Children and Adolescents Eye Study, stratified by sex, region and age………………………………………………………..…..3

**Supplementary eTable 3.** Proportion of individuals with suboptimal uncorrected distance visual acuity (UDVA) in the total population, and the proportion of myopic individuals using vision correction (spectacles) as well as those with suboptimal presenting distance visual acuity (PDVA) in the myopic population, stratified by sex, region, and age in the Wuhu Children and Adolescents Eye Study…..…4

**Supplementary eTable 4.** The percentiles of axial length in the Wuhu Children and Adolescents Eye Study, stratified by age and sex…………………………………………………………………………6

**Supplementary eTable 5.** Prevalence of fundus lesions in the Wuhu Children and Adolescents Eye Study, stratified by the level of education………………………………………………………………………7

**Supplementary eTable 6.** Prevalence of fundus lesions in the Wuhu Children and Adolescents Eye Study, stratified by sex and region………………………………………………………………………………8

**Supplementary eTable 7** Comparison of Demographic Characteristics Between Excluded and Included Participants…………………………………………………………...…………………………….........9

**Supplementary eTable 8** Results of Univariate and Multivariate Logistic Analyses…………………10

**Section S2: Supplementary eFigure 1-3**

**Supplementary eFigure 1**. Geographical location of Wuhu City and the distribution of schools by level across districts and counties, as included in the Wuhu Children and Adolescents Eye Study………….16

**Supplementary eFigure 2**. Prevalence of fundus tessellation in the Wuhu Children and Adolescents Eye Study stratified by axial length and sex……………………………………………………………17

**Supplementary eFigure3** - eFigure 3 Representative examples of fundus abnormalities： (A) Normal fundus； (B) Fundus tessellation；(C) Abnormally high cup-to-disc diameter ratio (>0.6); (D) Epiretinal membrane; (E) Retinal detachment; (F) Pathological myopia with diffuse, mostly peripapillary, chorioretinal atrophy. …………………………………………………….……………………………18

**Section S1: eTable**

**eTable 1. Gross Regional Product（****GDP）and Resident Population of Wuhu City by District and County, 2023**

| **Region** | **GDP (billion, RMB)** | **GDP (billion, US$)** | **Resident Population (thousand)** |
| --- | --- | --- | --- |
| Yijiang Distrct | 56.31 | 8.11 | 438.2 |
| Jinghu Distrct | 86.41 | 12.44 | 487.3 |
| Wanzhi Distrct | 41.37 | 5.96 | 352.4 |
| Fanchang Distrct | 38.45 | 5.54 | 252.7 |
| Jiujiang Distrct | 58.46 | 8.42 | 556.2 |
| Wuwei County-level city | 65.01 | 9.36 | 829.1 |
| Nanling County | 35.49 | 5.11 | 438.6 |

|  | **Number** | **Refractive error (diopters), median (quartile 1, 3)** | **Likely myopia (95% confidence interval (CI))** | **Myopia (95%CI)** | **High myopia (95%CI)** |
| --- | --- | --- | --- | --- | --- |
| Sex |  |  |  |  |  |
| Male | 162,366 | -1.125 (-2.75, -0.25) | 14.13% (13.96% - 14.30%) | 54.47% (54.22% - 54.71%) | 4.13% (4.04% - 4.23%) |
| Female | 140,370 | -1.375 (-3.00, -0.375) | 12.94% (12.76% - 13.11%) | 59.76% (59.50% - 60.02%) | 4.51% (4.40% - 4.62%) |
| Region |  |  |  |  |  |
| Urban area | 283,953 | -1.25 (-2.875, -0.25) | 13.53% (13.40% - 13.66%) | 56.93% (56.75% - 57.12%) | 4.32% (4.25% - 4.40%) |
| Rural area | 18,783 | -1.20 (-2.75, -0.375) | 14.27% (13.78% - 14.77%) | 56.70% (56.00% - 57.40%) | 4.12% (3.83% - 4.41%) |
| Age (years) |  | | | | |
| ≤3 | 492 | -0.125 (-0.625, 0.25) | 12.40% (9.55% - 15.45%) | 17.28% (14.02% - 20.73%) | 0.00% (0.00% - 0.00%) |
| 4 | 2,057 | -0.125 (-0.50, 0.375) | 14.29% (12.83% - 15.80%) | 14.78% (13.32% - 16.33%) | 0.53% (0.24% - 0.88%) |
| 5 | 2,298 | -0.125 (-0.50, 0.25) | 13.93% (12.58% - 15.32%) | 14.62% (13.23% - 16.10%) | 0.22% (0.04% - 0.44%) |
| 6 | 16,178 | -0.25 (-0.75, 0.125) | 17.22% (16.63% - 17.80%) | 19.42% (18.82% - 20.04%) | 0.35% (0.26% - 0.44%) |
| 7 | 30,981 | -0.25 (-0.875, 0.00) | 18.01% (17.58% - 18.45%) | 24.09% (23.60% - 24.56%) | 0.32% (0.26% - 0.39%) |
| 8 | 34,025 | -0.50 (-1.25, 0.125) | 18.56% (18.15% - 18.98%) | 34.01% (33.51% - 34.51%) | 0.46% (0.39% - 0.53%) |
| 9 | 32,522 | -0.75 (-1.875, 0.25) | 17.24% (16.84% - 17.64%) | 45.88% (45.34% - 46.43%) | 0.96% (0.85% - 1.07%) |
| 10 | 34,363 | -1.125 (-2.375, 0.375) | 15.93% (15.55% - 16.31%) | 55.93% (55.43% - 56.46%) | 1.69% (1.56% - 1.83%) |
| 11 | 31,937 | -1.50 (-3.00, 0.50) | 13.53% (13.17% - 13.90%) | 64.63% (64.10% - 65.15%) | 3.12% (2.93% - 3.32%) |
| 12 | 29,424 | -2.00 (-3.50, 0.75) | 11.67% (11.30% - 12.04%) | 72.06% (71.56% - 72.58%) | 5.02% (4.77% - 5.27%) |
| 13 | 29,446 | -2.375 (-4.00, 1.125) | 9.48% (9.14% - 9.82%) | 78.32% (77.83% - 78.79%) | 7.39% (7.09% - 7.69%) |
| 14 | 28,745 | -2.75 (-4.375, 1.375) | 7.97% (7.66% - 8.28%) | 83.02% (82.60% - 83.45%) | 9.94% (9.58% - 10.28%) |
| 15 | 19,015 | -3.00 (-4.625, 1.625) | 7.08% (6.73% - 7.45%) | 85.49% (84.97% - 85.98%) | 12.16% (11.71% - 12.63%) |
| 16 | 5,835 | -3.50 (-5.125, 2.00) | 4.85% (4.30% - 5.42%) | 89.46% (88.69% - 90.25%) | 15.48% (14.57% - 16.40%) |
| 17 | 3,810 | -3.875 (-5.50, 2.375) | 3.91% (3.33% - 4.54%) | 92.26% (91.42% - 93.10%) | 19.63% (18.43% - 20.89%) |
| ≥18 | 1,608 | -4.00 (-5.625, 2.75) | 3.11% (2.30% - 3.98%) | 94.28% (93.10% - 95.40%) | 21.64% (19.65% - 23.69%) |

**eTable 2. Prevalence of myopia and high myopia in the Wuhu Children and Adolescents Eye Study, stratified by sex, region and age**

Note: Likely myopia: refractive error >-1.00 diopter (D) and ≤−0.50 D; myopia: refractive error ≤−1.00 D; low myopia: -3.00 D < refractive error ≤-1.00 D; moderate myopia: -6.00 D < refractive error ≤-3.00 D; high myopia: refractive error ≤-6.00 D

**eTable 3.** **Proportion of individuals with suboptimal uncorrected distance visual acuity (UDVA) in the total population, and the proportion of myopic individuals using vision correction (spectacles) as well as those with suboptimal presenting distance visual acuity (PDVA) in the myopic population, stratified by sex, region, and age in the Wuhu Children and Adolescents Eye Study.**

|  | **Total number of individuals** | **Percentage of individuals with suboptimal UDVA** | **Number of myopic individuals** | **Number of myopic individuals using vision correction** | **Percentage of myopic individuals using vision correction** | **Percentage of suboptimal PDVA in myopic population** |
| --- | --- | --- | --- | --- | --- | --- |
| Total | 302,736 | 64.83% (64.67% - 65.00%) | 172,317 | 82803 | 48.05% (47.81% - 48.28%) | 65.84% (65.51% - 66.16%) |
| Sex |  |  |  |  |  |  |
| Male | 162,366 | 62.27% (62.04% - 62.51%) | 88,436 | 40441 | 45.73% (45.41% - 46.05%) | 64.77% (64.30% - 65.23%) |
| Female | 140,370 | 67.79% (67.55% - 68.04%) | 83,881 | 42362 | 50.50% (50.17% - 50.84%) | 66.86% (66.42% - 67.30%) |
| Region |  |  |  |  |  |  |
| Urban area | 283,953 | 64.92% (64.75% - 65.10%) | 161,667 | 78,806 | 48.75% (48.51% - 49.00%) | 65.60% (65.28% - 65.93%) |
| Rural area | 18,783 | 63.45% (62.76% - 64.14%) | 10,650 | 3,997 | 37.53% (36.63% - 38.44%) | 70.65% (69.25% - 72.08%) |
| Age (years) |  |  |  | | | |
| ≤3 | 492 | 4.67% (2.85% - 6.71%) | 85 | 2 | 2.35% (0.00% - 5.88%) | 0.00% (0.00% - 0.00%) |
| 4 | 2,057 | 12.55% (11.14% - 14.01%) | 304 | 8 | 2.63% (0.99% - 4.61%) | 25.00% (0.00% - 62.50%) |
| 5 | 2,298 | 63.49% (61.49% - 65.45%) | 336 | 12 | 3.57% (1.79% - 5.65%) | 83.33% (58.33% - 100.00%) |
| 6 | 16,178 | 41.78% (41.03% - 42.55%) | 3,142 | 310 | 9.87% (8.85% - 10.95%) | 63.55% (58.06% - 69.03%) |
| 7 | 30,981 | 41.49% (40.94% - 42.04%) | 7,462 | 973 | 13.04% (12.30% - 13.80%) | 65.67% (62.59% - 68.65%) |
| 8 | 34,025 | 47.09% (46.57% - 47.63%) | 11,572 | 2515 | 21.73% (20.98% - 22.49%) | 63.78% (61.83% - 65.65%) |
| 9 | 32,522 | 54.40% (53.85% - 54.94%) | 14,921 | 4534 | 30.39% (29.63% - 31.10%) | 64.36% (62.92% - 65.79%) |
| 10 | 34,363 | 62.48% (61.96% - 62.99%) | 19,220 | 7366 | 38.32% (37.64% - 39.01%) | 64.89% (63.79% - 65.94%) |
| 11 | 31,937 | 69.41% (68.92% - 69.90%) | 20,641 | 9114 | 44.15% (43.48% - 44.82%) | 65.50% (64.54% - 66.52%) |
| 12 | 29,424 | 75.60% (75.11% - 76.09%) | 21,202 | 10908 | 51.45% (50.78% - 52.11%) | 64.60% (63.72% - 65.52%) |
| 13 | 29,446 | 81.18% (80.72% - 81.63%) | 23,061 | 13516 | 58.61% (57.98% - 59.24%) | 64.31% (63.51% - 65.10%) |
| 14 | 28,745 | 84.95% (84.53% - 85.37%) | 23,865 | 14965 | 62.71% (62.10% - 63.32%) | 66.14% (65.37% - 66.91%) |
| 15 | 19,015 | 87.22% (86.72% - 87.70%) | 16,255 | 10686 | 65.74% (65.01% - 66.47%) | 67.42% (66.54% - 68.29%) |
| 16 | 5,835 | 91.34% (90.61% - 92.05%) | 5,220 | 3874 | 74.21% (73.01% - 75.38%) | 68.02% (66.60% - 69.44%) |
| 17 | 3,810 | 93.38% (92.57% - 94.17%) | 3,515 | 2816 | 80.11% (78.75% - 81.45%) | 70.49% (68.79% - 72.16%) |
| ≥18 | 1,608 | 94.65% (93.53% - 95.71%) | 1,516 | 1204 | 79.42% (77.37% - 81.46%) | 77.91% (75.58% - 80.23%) |

**eTable 4. The percentiles of axial length in the Wuhu Children and Adolescents Eye Study, stratified by age and sex**

| **Age (years)** | ***P*_5_** | ***P_10_*** | ***P*_25_** | ***P*_50_** | ***P*_75_** | ***P*_90_** | ***P*_95_** | ***P*_5_** | ***P_10_*** | ***P*_25_** | ***P*_50_** | ***P*_75_** | ***P*_90_** | ***P*_95_** |
| --- | --- | --- | --- | --- | --- | --- | --- | --- | --- | --- | --- | --- | --- | --- |
|  | **Male** | | | | | | | **Female** | | | | | | |
| 6 | 21.89 | 22.22 | 22.68 | 23.15 | 23.62 | 24.05 | 24.35 | 21.30 | 21.71 | 22.15 | 22.59 | 23.06 | 23.10 | 23.77 |
| 7 | 22.00 | 22.36 | 22.80 | 23.28 | 23.79 | 24.26 | 24.59 | 21.43 | 21.82 | 22.29 | 22.76 | 23.25 | 23.50 | 24.05 |
| 8 | 22.26 | 22.57 | 23.05 | 23.58 | 24.14 | 24.70 | 25.08 | 21.74 | 22.08 | 22.57 | 23.09 | 23.63 | 23.74 | 24.59 |
| 9 | 22.48 | 22.78 | 23.27 | 23.84 | 24.49 | 25.14 | 25.57 | 22.05 | 22.35 | 22.84 | 23.41 | 24.05 | 24.19 | 25.05 |
| 10 | 22.64 | 22.97 | 23.48 | 24.12 | 24.84 | 25.52 | 25.93 | 22.22 | 22.54 | 23.07 | 23.72 | 24.40 | 24.67 | 25.44 |
| 11 | 22.77 | 23.12 | 23.67 | 24.35 | 25.14 | 25.82 | 26.23 | 22.35 | 22.71 | 23.29 | 23.97 | 24.70 | 25.04 | 25.77 |
| 12 | 22.90 | 23.24 | 23.84 | 24.59 | 25.41 | 26.13 | 26.52 | 22.49 | 22.87 | 23.48 | 24.22 | 24.94 | 25.38 | 26.05 |
| 13 | 23.03 | 23.41 | 24.05 | 24.82 | 25.65 | 26.37 | 26.80 | 22.60 | 23.00 | 23.66 | 24.39 | 25.13 | 25.61 | 26.20 |
| 14 | 23.07 | 23.49 | 24.18 | 25.00 | 25.85 | 26.60 | 27.08 | 22.74 | 23.12 | 23.77 | 24.53 | 25.27 | 25.81 | 26.38 |
| 15 | 23.15 | 23.55 | 24.26 | 25.12 | 25.99 | 26.74 | 27.19 | 22.75 | 23.17 | 23.87 | 24.63 | 25.40 | 25.98 | 26.53 |
| 16 | 23.21 | 23.68 | 24.43 | 25.36 | 26.25 | 26.97 | 27.41 | 22.81 | 23.28 | 24.05 | 24.86 | 25.65 | 26.12 | 26.90 |
| 17 | 23.39 | 23.87 | 24.70 | 25.60 | 26.44 | 27.14 | 27.59 | 22.97 | 23.48 | 24.22 | 24.97 | 25.73 | 26.38 | 26.82 |
| ≥18 | 23.58 | 24.02 | 24.79 | 25.65 | 26.40 | 27.20 | 27.74 | 22.97 | 23.42 | 24.18 | 24.95 | 25.73 | 26.40 | 27.04 |

-

| **Education phase** | **School grade** | **Number** | **Fundus Tessellation (95% confidence interval (CI))** | **Increased cup-to-disc ratio (95% CI)** | **Others (95% CI)** |
| --- | --- | --- | --- | --- | --- |
| Elementary school | 4 | 32,702 | 3.03% (2.84% - 3.22%) | 2.11% (1.95% - 2.26%) | 0.0031% (0.0000% - 0.0092%) |
|  | 5 | 31,800 | 3.76% (3.55% - 3.97%) | 2.07% (1.91% - 2.22%) | 0.0157% (0.0031% - 0.0314%) |
|  | 6 | 32,031 | 4.68% (4.45% - 4.91%) | 1.93% (1.78% - 2.08%) | 0.0125% (0.0031% - 0.0250%) |
|  | 7 | 31,956 | 5.90% (5.64% - 6.17%) | 1.70% (1.56% - 1.84%) | 0.0063% (0.0000% - 0.0156%) |
|  | 8 | 33,119 | 7.12% (6.85% - 7.39%) | 1.59% (1.46% - 1.73%) | 0.0030% (0.0000% - 0.0091%) |
|  | 9 | 29,668 | 9.07% (8.74% - 9.40%) | 1.47% (1.34% - 1.61%) | 0.0135% (0.0034% - 0.0270%) |
|  | Total | 191,276 | 5.55% (5.45% - 5.65%) | 1.82% (1.76% - 1.87%) | 0.0089% (0.0047% - 0.0131%) |
| Middle school | 10 | 28,814 | 11.22% (10.85% - 11.57%) | 1.60% (1.46% - 1.75%) | 0.0104% (0.0000% - 0.0243%) |
|  | 11 | 28,635 | 13.54% (13.14% - 13.94%) | 1.57% (1.43% - 1.71%) | 0.0105% (0.0000% - 0.0244%) |
|  | 12 | 26,667 | 15.81% (15.37% - 16.24%) | 1.54% (1.40% - 1.69%) | 0.0225% (0.0075% - 0.0412%) |
|  | Total | 84,116 | 13.46% (13.24% - 13.69%) | 1.57% (1.49% - 1.66%) | 0.0143% (0.0071% - 0.0226%) |
| High school | 13 | 4,730 | 8.88% (8.08% - 9.70%) | 1.33% (1.01% - 1.67%) | 0.0211% (0.0000% - 0.0634%) |
|  | 14 | 4,393 | 10.61% (9.74% - 11.54%) | 1.55% (1.21% - 1.93%) | 0.1366% (0.0455% - 0.2504%) |
|  | 15 | 2,343 | 13.40% (11.99% - 14.77%) | 1.37% (0.94% - 1.84%) | 0.0000% (0.0000% - 0.0000%) |
|  | Total | 11,466 | 10.47% (9.91% - 11.04%) | 1.42% (1.21% - 1.64%) | 0.0611% (0.0174% - 0.1134%) |

**eTable 5. Prevalence of fundus lesions in the Wuhu Children and Adolescents Eye Study, stratified by the level of education**

| **Funds Lesion** | **Number** | **Total prevalence (95% CI)** | **Male Prevalence (95% CI)** | **Female Prevalence (95% CI)** | **Urban Prevalence (95% CI)** | **Rural Prevalence (95% CI)** |
| --- | --- | --- | --- | --- | --- | --- |
| Fundus tessellation | 22,548 | 7959.4 (7860.5 - 8056.8) | 8505.8 (8365.5 - 8643.4) | 7328.0 (7183.4 - 7471.0) | 8185.1 (8081.2 - 8289.7) | 4556.5 (4255.8 - 4880.0) |
| Large cup-to-disc diameter ratio | 4,915 | 1735.0 (1687.3 - 1783.7) | 2096.7 (2024.9 - 2169.1) | 1317.1 (1257.0 - 1377.9) | 1725.9 (1677.7 - 1775.5) | 1872.6 (1673.9 - 2076.8) |
| Epiretinal membranes | 19 | 6.7 (3.9 - 9.9) | 7.9 (4.0 - 12.5) | 5.3 (1.5 - 9.1) | 6.8 (3.8 - 9.8) | 5.7 (0.0 - 17.0) |
| Retinal detachment | 5 | 1.8 (0.4 - 3.5) | 2.6 (0.7 - 5.3) | 0.8 (0.0 - 2.3) | 1.9 (0.4 - 3.8) | 0.0 (0.0 - 0.0) |
| Pathological myopia | 4 | 1.4 (0.4 - 2.8) | 1.3 (0.0 - 3.3) | 1.5 (0.0 - 3.8) | 1.1 (0.0 - 2.6) | 5.7 (0.0 - 17.0) |
| Macular hole | 3 | 1.1 (0.0 - 2.5) | 1.3 (0.0 - 3.3) | 0.8 (0.0 - 2.3) | 0.8 (0.0 - 1.9) | 5.7 (0.0 - 17.0) |
| Retinal vein occlusions | 3 | 1.1 (0.0 - 2.5) | 1.3 (0.0 - 3.3) | 0.8 (0.0 - 2.3) | 1.1 (0.0 - 2.6) | 0.0 (0.0 - 0.0) |
| Diabetic retinopathy | 1 | 0.4 (0.0 - 1.1) | 0.7 (0.0 - 2.0) | 0.0 (0.0 - 0.0) | 0.4 (0.0 - 1.1) | 0.0 (0.0 - 0.0) |
| Optic disc edema | 1 | 0.4 (0.0 - 1.1) | 0.0 (0.0 - 0.0) | 0.8 (0.0 - 2.3) | 0.4 (0.0 - 1.1) | 0.0 (0.0 - 0.0) |
| Retinal arteriosclerosis | 19 | 6.7 (3.9 - 9.9) | 5.9 (2.6 - 9.9) | 7.6 (3.0 - 12.9) | 6.4 (3.4 - 9.8) | 11.3 (0.0 - 28.4) |
| Optic atrophy | 3 | 1.1 (0.0 - 2.5) | 1.3 (0.0 - 3.3) | 0.8 (0.0 - 2.3) | 1.1 (0.0 - 2.6) | 0.0 (0.0 - 0.0) |

**eTable 6. Prevalence of fundus lesions in the Wuhu Children and Adolescents Eye Study, stratified by sex and region**

Prevalence unit: per 100,000 individuals.

eTable7 Comparison of Demographic Characteristics Between Excluded and Included Participants

| Characteristics | Participants excluded from the statustical analysis | | | Participants included into the statistical analysis | P-value* |
| --- | --- | --- | --- | --- | --- |
|  | Total exclusion | Participants with inaccurate personal information or inconsistent examination records | Participants Wearing ortho-K or lack of SE data |  |  |
| Number | 12, 833 | 7,251 | 5,582 | 302, 736 |  |
| Age (years, mean ± SD) | 10.7 ± 12.9 | 10.8 ± 16.9 | 10.6 ± 2.8 | 10.6 ± 2.9 | <0.001 |
| Sex (male, %) | 7,040 (54.86%) | 4,193 (57.83%) | 2,847 (51.00%) | 162,366 (53.63%) | 0.004 |
| Residence (Urban, %) | 12,256 (95.50%) | 6,727 (92.77%) | 5,529 (99.05%) | 283,953 (93.80%) | <0.001 |

* Comparison of Demographics and Characteristics: Included vs. Excluded Participants

eTable8 Results of Univariate and Multivariate Logistic Analyses

|  | Univariate Analysis | | | | | Multivariate Analysis | | | | | |
| --- | --- | --- | --- | --- | --- | --- | --- | --- | --- | --- | --- |
| **Variables** | **Coefficient (β)** | **Standard error of β** | **Wald χ²** | **OR (95CI%)** | **P-value** | **Variables** | **Coefficient (β)** | **Standard error of β** | **Wald χ²** | **OR (95CI%)** | **P-value** |
| **prevalence of myopia** |  | | | | | | | | | | |
| Gender (male=1) | -0.216 | 0.007 | 858.261 | 0.806 (0.794 - 0.817) | <0.001 | Gender  (male=1) | -0.988 | 0.11 | 8720.274 | 0.372 (0.365- 0.380) | <0.001 |
| Age | 0.374 | 0.002 | 52005.640 | 1.454 (1.449 – 1.459) | <0.001 | age | 0.185 | 0.002 | 8117.554 | 1.203 (1.198 - 1.208) | <0.001 |
| School grade | 0.390 | 0.002 | 53638.882 | 1.477 (1.472 – 1.482) | <0.001 | Axial length | 1.310 | 0.006 | 40791.013 | 3.707 (3.660 - 3.754) | <0.001 |
| Axial length | 1.365 | 0.006 | 60656.017 | 3.914 (3.872 – 3.957) | <0.001 | Fundus tessellation (Postive=1) | 0.094 | 0.021 | 19.866 | 1.099 (1.054 - 1.146) | <0.001 |
| Region (Urban=1) | 0.010 | 0.015 | 0.394 | 1.010 (0.980 – 1.040) | 0.530 | Region (Urban=1) | 0.057 | 0.020 | 7.847 | 1.059 (1.017 – 1.102) | <0.001 |
| AL/CRC | 14.684 | 0.057 | 65849.349 | *2383360.210 (2130499.954 – 2666231.406)* | <0.001 |  |  |  |  |  |  |
| Fundus tessellation (Postive=1) | 1.021 | 0.017 | 3777.761 | 2.776 (2.687 – 2.868) | <0.001 |  |  |  |  |  |  |
| **prevalence of likely myopia** |  | | | | | | | | | | |
| Gender (male=1) | 0.102 | 0.011 | 92.089 | 1.108 (1.085 - 1.131) | <0.001 | Gender (male=1) | 0.321 | 0.012 | 732.588 | 1.379 (1.347 – 1.411) | <0.001 |
| Age | -0.120 | 0.002 | 4046.021 | 0.887 (0.884 - 0.890) | <0.001 | age | -0.040 | 0.002 | 278.284 | 0.961 (0.956 – 0.965) | <0.001 |
| School grade | -0.124 | 0.002 | 4143.818 | 0.883 (0.880 – 0.887) | <0.001 | axial length | -0.437 | 0.006 | 5428.423 | 0.646 (0.638 – 0.653) | 0.000 |
| Axial length | -0.457 | 0.005 | 8749.024 | 0.633 (0.627 – 0.639) | <0.001 | Region (Urban=1) | -0.068 | 0.023 | 8.296 | 0.935 (0.893 – 0.979) | 0.004 |
| Region (Urban=1) | -0.062 | 0.022 | 8.258 | 0.940 (0.901 – 0.980) | 0.004 | Fundus tessellation (Postive=1) | -0.164 | 0.026 | 39.862 | 0.849 (0.807 – 0.893) | <0.001 |
| AL/CRC | -4.356 | 0.043 | 10473.850 | 0.013 (0.012 – 0.014) | <0.001 |  |  |  |  |  |  |
| Fundus tessellation (Postive=1) | -0.596 | 0.025 | 573.692 | 0.551 (0.525 – 0.579) | <0.001 |  |  |  |  |  |  |
| **prevalence of high myopia** |  | | | | | | | | | | |
| Gender (male=1) | -0.093 | 0.018 | 27.034 | 0.911 (0.880 – 0.944) | <0.001 | Gender (male=1) | -1.137 | 0.024 | 2215.673 | 0.321 (0.306 – 0.336) | <0.001 |
| Age | 0.394 | 0.004 | 11336.486 | 1.483 (1.472 – 1.494) | <0.001 | age | 0.109 | 0.005 | 429.008 | 1.115 (1.104 – 1.127) | <0.001 |
| School grade | 0.414 | 0.004 | 11575.654 | 1.513 (1.502 – 1.525) | <0.001 | axial length | 1.773 | 0.014 | 16678.924 | 5.889 (5.733 – 6.050) | <0.001 |
| Axial length | 1.719 | 0.012 | 22208.261 | 5.580 (5.456 – 5.708) | <0.001 | Fundus tessellation (Postive=1) | 0.189 | 0.029 | 43.436 | 1.208 (1.142 – 1.277) | <0.001 |
| Region (Urban=1) | 0.049 | 0.038 | 1.692 | 1.050 (0.975 – 1.131) | 0.193 |  |  |  |  |  |  |
| AL/CRC | 18.537 | 0.129 | 20784.818 | 112357255.7 (87328151.54 – 144559946.5) | <0.001 |  |  |  |  |  |  |
| Fundus tessellation (Postive=1) | 1.383 | 0.022 | 3845.072 | 3.986 (3.815 – 4.164) | <0.001 |  |  |  |  |  |  |
| **Prevalence of suboptimal UDVA** |  | | | | | | | | | | |
| Gender (male=1) | -0.241 | 0.008 | 992.565 | 0.786 (0.774 – 0.798) | <0.001 | Gender (male=1) | -0.495 | 0.010 | 2242.324 | 0.609 (0.597 – 0.622) | <0.001 |
| Age | 0.280 | 0.002 | 33238.726 | 1.323 (1.320 – 1.327) | <0.001 | age | 0.081 | 0.002 | 1524.214 | 1.085 (1.080 – 1.089) | <0.001 |
| School grade | 0.290 | 0.002 | 34059.521 | 1.336 (1.332 – 1.340) | <0.001 | axial length | 0.511 | 0.006 | 7417.665 | 1.667 (1.648 – 1.686) | <0.001 |
| Axial length | 0.926 | 0.005 | 41668.484 | 2.525 (2.503 – 2.548) | <0.001 | Region (Urban=1) | 0.133 | 0.020 | 42.314 | 1.142 (1.097 – 1.188) | <0.001 |
| Region (Urban=1) | 0.063 | 0.016 | 16.153 | 1.065 (1.033 – 1.098) | <0.001 | Fundus tessellation (Postive=1) | 0.184 | 0.022 | 71.617 | 1.202 (1.152 – 1.254) | <0.001 |
| AL/CRC | 10.295 | 0.046 | 50531.024 | 29584.880 (27044.959 – 32363.335) | <0.001 | Mopia (Postive=1) | 1.803 | 0.011 | 26218.103 | 6.070 (5.939 – 6.204) | <0.001 |
| Fundus tessellation (Postive=1) | 0.924 | 0.018 | 2714.314 | 2.520 (2.434 – 2.609) | <0.001 |  |  |  |  |  |  |
| Mopia (Postive=1) | 2.482 | 0.009 | 72989.268 | 11.970 (11.757 – 12.188) | <0.001 |  |  |  |  |  |  |
| **Prevalence of suboptimal PDVA in the myopic population** |  | | | | | | | | | | |
| Gender (male=1) | -0.192 | 0.010 | 341.673 | 0.826 (0.809 – 0.843) | <0.001 | Gender (male=1) | -0.709 | 0.013 | 3156.056 | 0.492 (0.480 – 0.505) | <0.001 |
| Age | 0.223 | 0.002 | 10688.668 | 1.250 (1.245 – 1.255) | <0.001 | age | 0.111 | 0.003 | 1855.970 | 1.117 (1.111 - 1.123) | <0.001 |
| School grade | 0.235 | 0.002 | 11139.762 | 1.265 (1.260 – 1.271) | <0.001 | axial length | 0.799 | 0.007 | 14580.694 | 2.223 (2.194 – 2.252) | <0.001 |
| Axial length | 0.797 | 0.006 | 19848.549 | 2.219 (2.194 – 2.244) | <0.001 | Region (Urban=1) | 0.271 | 0.026 | 107.859 | 1.312 (1.246 – 1.381) | <0.001 |
| Region (Urban=1) | 0.264 | 0.023 | 137.049 | 1.303 (1.246 – 1.362) | <0.001 | Fundus tessellation (Postive=1) | 0.065 | 0.018 | 12.386 | 1.067 (1.029 – 1.106) | <0.001 |
| AL/CRC | 8.189 | 0.052 | 24663.406 | 3601.123 (3251.269 – 3988.624) | <0.001 |  |  |  |  |  |  |
| Fundus tessellation (Postive=1) | 0.624 | 0.016 | 1487.551 | 1.867 (1.809 – 1.927) | <0.001 |  |  |  |  |  |  |
| **prevalence of high axial myopia** |  | | | | | | | | | | |
| Gender (male=1) | 0.868 | 0.016 | 2928.895 | 2.382 (2.308 – 2.458) | <0.001 | Gender (male=1) | 1.087 | 0.021 | 2804.175 | 2.965 (2.848 – 3.086) | <0.001 |
| Age | 0.410 | 0.003 | 17322.078 | 1.507 (1.498 – 1.516) | <0.001 | age | 0.170 | 0.004 | 1565.969 | 1.185 (1.175 – 1.195) | <0.001 |
| School grade | 0.433 | 0.003 | 17763.651 | 1.542 (1.533 – 1.552) | <0.001 | Region (Urban=1) | 0.111 | 0.042 | 7.008 | 1.117 (1.029 – 1.213) | 0.008 |
| Region (Urban=1) | 0.185 | 0.033 | 31.859 | 1.203 (1.128 – 1.283) | <0.001 | Fundus tessellation (Postive=1) | 0.768 | 0.024 | 1005.230 | 2.156 (2.056 – 2.261) | <0.001 |
| Fundus tessellation (Postive=1) | 1.479 | 0.018 | 6561.125 | 4.387 (4.233 – 4.547) | <0.001 |  |  |  |  |  |  |
| **Prevalence of fundus tesellation** |  | | | | | | | | | | |
| Gender (male=1) | 0.156 | 0.014 | 126.318 | 1.168 (1.137 – 1.200) | <0.001 | Gender (male=1) | 0.197 | 0.014 | 191.692 | 1.218 (1.185 – 1.253) | <0.001 |
| Age | 0.176 | 0.002 | 5105.278 | 1.193 (1.187 – 1.199) | <0.001 | age | 0.132 | 0.003 | 2333.261 | 1.141 (1.135 - 1.148) | <0.001 |
| School grade | 0.187 | 0.003 | 5433.013 | 1.205 (1.199 – 1.211) | <0.001 | Region (Urban=1) | 0.682 | 0.037 | 337.924 | 1.977 (1.838 - 2.126) | <0.001 |
| Axial length | 0.600 | 0.006 | 11635.854 | 1.823 (1.803 – 1.843) | <0.001 | Myopia (Postiev=1) | 0.702 | 0.018 | 1519.283 | 2.018 (1.948 – 2.091) | <0.001 |
| Region (Urban=1) | 0.638 | 0.037 | 301.346 | 1.893 (1.761 – 2.034) | <0.001 |  |  |  |  |  |  |
| AL/CRC | 3.873 | 0.044 | 7659.196 | 48.068 (44.075 – 52.423) | <0.001 |  |  |  |  |  |  |
| Myopia (Postiev=1) | 1.021 | 0.017 | 3777.761 | 2.776 (2.687 – 2.868) | <0.001 |  |  |  |  |  |  |
| **Prevalence of an abnormally high cup-to-disc diameter ratio** |  | | | | | | | | | | |
| Gender (male=1) | 0.480 | 0.030 | 255.724 | 1.615 (1.523 – 1.713) | <0.001 | Gender (male=1) | 0.381 | 0.032 | 140.787 | 1.464 (1.375 - 1.559) | <0.001 |
| Age | -0.041 | 0.005 | 63.239 | 0.960 (0.950 – 0.970) | <0.001 | age | -0.060 | 0.007 | 85.405 | 0.942 (0.930 - 0.954) | <0.001 |
| School grade | -0.044 | 0.005 | 70.831 | 0.957 (0.947 – 0.967) | <0.001 | axial length | 0.139 | 0.016 | 73.522 | 1.149 (1.113 - 1.186) | <0.001 |
| Axial length | 0.059 | 0.012 | 26.507 | 1.061 (1.037 – 1.085) | <0.001 | Myopia (Postiev=1) | -0.370 | 0.038 | 95.948 | 0.691 (0.642 - 0.744) | <0.001 |
| Region (Urban=1) | -0.086 | 0.058 | 2.224 | 0.918 (0.820 – 1.027) | 0.136 | Fundus tessellation (Postive=1) | 0.752 | 0.043 | 304.531 | 2.122 (1.950 - 2.309) | <0.001 |
| AL/CRC | -1.223 | 0.101 | 147.139 | 0.294 (0.242 – 0.359) | <0.001 |  |  |  |  |  |  |
| Myopia (Postiev=1) | -0.275 | 0.029 | 90.851 | 0.759 (0.718 – 0.804) | <0.001 |  |  |  |  |  |  |
| Fundus tessellation (Postive=1) | 0.719 | 0.040 | 317.506 | 2.052 (1.896 – 2.220) | <0.001 |  |  |  |  |  |  |

**Section S2: Supplementary eFigure**


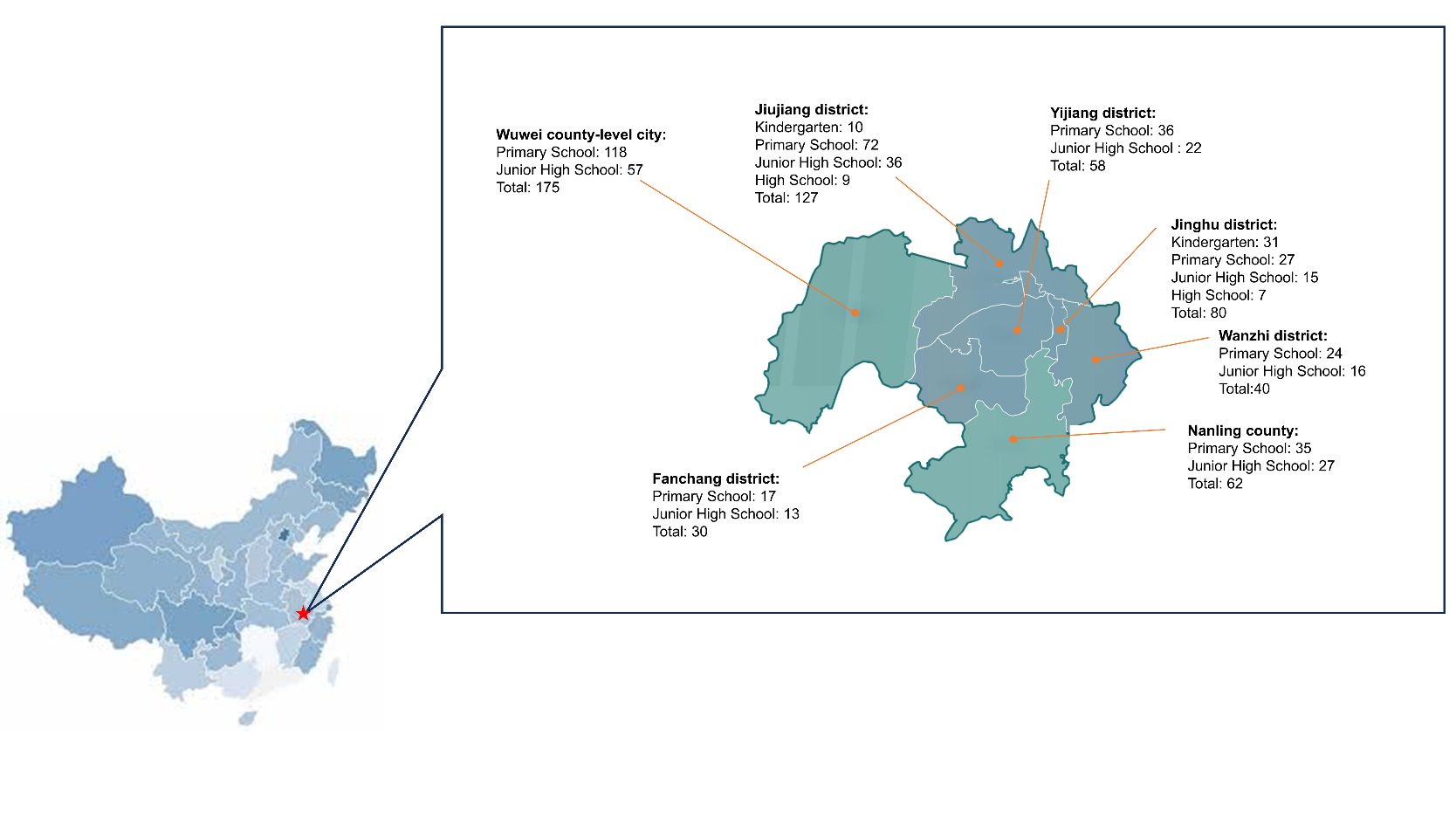


**eFigure1. Geographical location of Wuhu City and the distribution of schools by level across districts and counties, as included in the Wuhu Children and Adolescents Eye Study**

**eFigure 2. Prevalence of fundus tessellation in the Wuhu Children and Adolescents Eye Study stratified by axial length and sex**


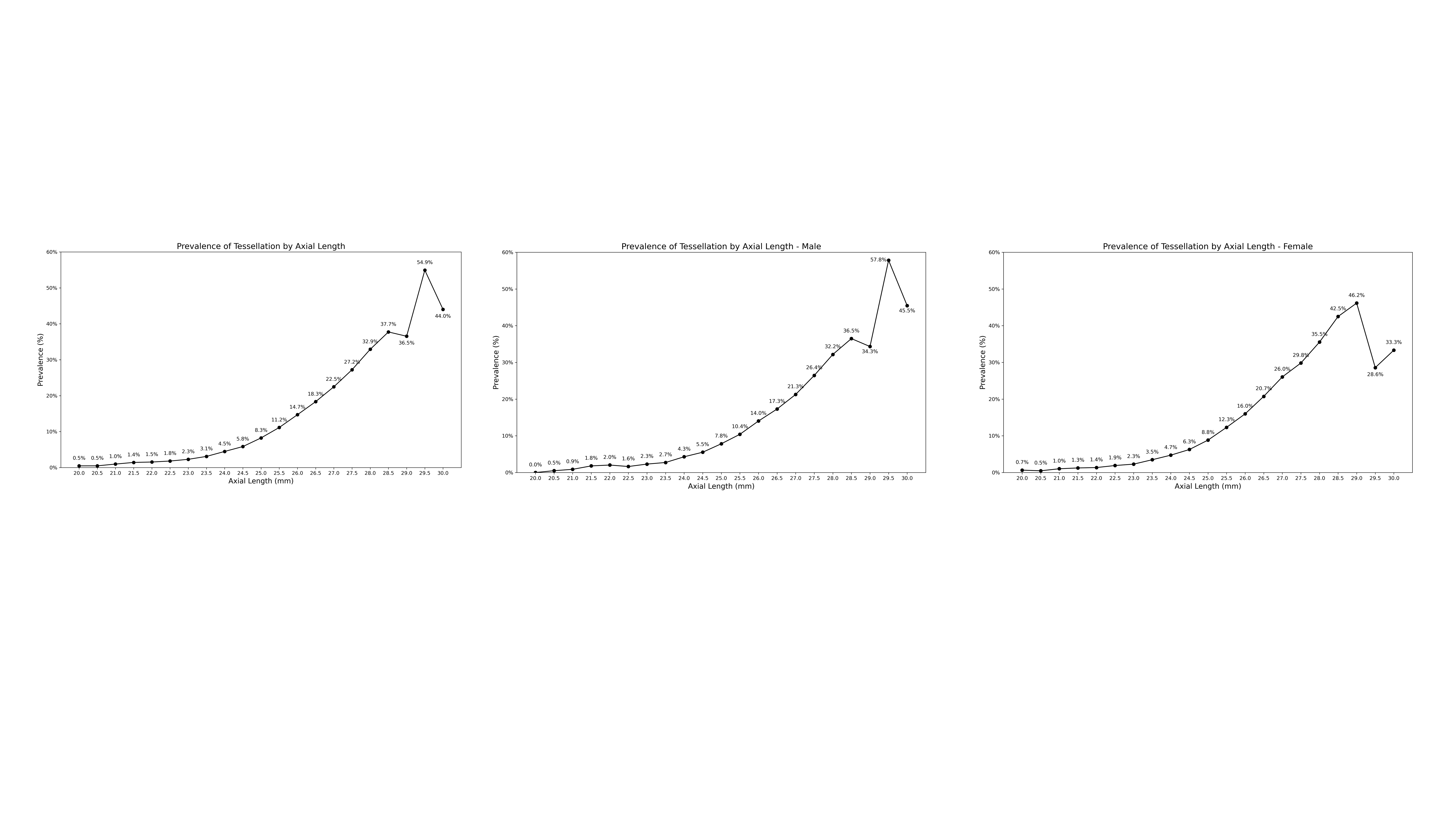


**eFigure3 Representative examples of fundus abnormalities： (A) Normal fundus; (B) Fundus tessellation; (C) Abnormally high cup-to-disc diameter ratio (>0.6); (D) Epiretinal membrane; (E) Retinal detachment; (F) Pathological myopia with diffuse, mostly peripapillary, chorioretinal atrophy**


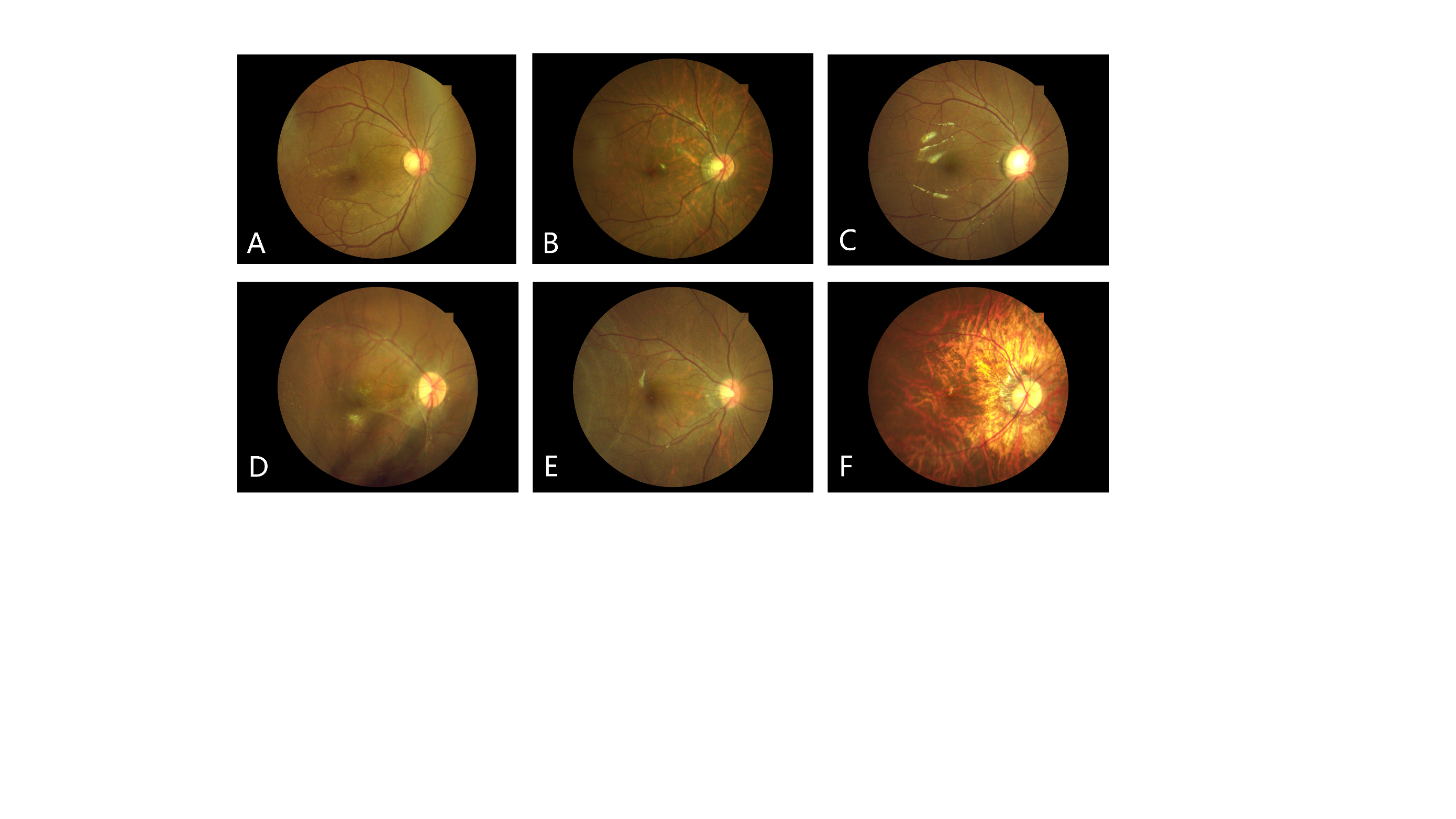

Supplement: Supplement 1 [file iovs-66-6-33_s001.docx]
